# Occurrence, Distribution and Ecological Risk of Bisphenol Analogues in the Surface Water from a Water Diversion Project in Nanjing, China

**DOI:** 10.3390/ijerph16183296

**Published:** 2019-09-07

**Authors:** Chaoya Zheng, Jianchao Liu, Jinghua Ren, Jie Shen, Jian Fan, Ruiyu Xi, Wei Chen, Qing Chen

**Affiliations:** 1 Key Laboratory of Integrated Regulation and Resources Development, College of Environment, Hohai University, Nanjing 210098, China (C.Z.) (R.X.) (W.C.); 2 Engineering Innovation Center of Land Ecological Monitoring and Remediation, Ministry of Natural Resources, Geological Survey of Jiangsu Province, Nanjing 210018, China (J.R.) (J.F.); 3 Everbright Environmental Protection Technology and Equipment (Changzhou) Co., Ltd., Changzhou 213011, China; 4 Suzhou Litree Ultra-Filtration Membrane Technology Co., Ltd., Suzhou 215000, China

**Keywords:** bisphenol analogues, colloids, suspended particulate matter, environmental risk, water diversion project

## Abstract

Due to the widespread use of bisphenol analogues (BPs) as alternatives to bisphenol A (BPA), considerable attention for health risk has been shown in aquatic ecosystems. The occurrence and distribution of six BPs were researched in a soluble phase (<10^−3^ μm), colloidal phase (10^−3^ μm to 1 µm), and suspended particulate matter (SPM >1 µm) in a water diversion project of Nanjing, China. Except for bisphenol Z, all BPs were detected in two or three phases, where the total concentrations of detected BPs were 161–613 ng/L, 5.19–77.2 ng/L, and 47.5–353 ng/g for the soluble phase, colloidal phase, and SPM, respectively. Among the detected compounds, BPA is still the dominant BPs in the soluble and colloidal phases, which is followed by bisphenol-S , while bisphenol-AF was the major contaminant in SPM, followed by BPA. The mean contribution proportions of colloids were 1–2 orders of magnitude higher than SPM, which suggests that colloids have a clear impact on regulating BPs’ environmental behaviors. In terms of spatial distribution, the water diversion project could reduce the pollution levels of BPs, which might further affect the ecological security of the Yangtze River.

## 1. Introduction

Environmental hormone bisphenol-A (2,2-bis(4-hydroxyphenyl) propane, BPA) has varying different adverse effects toward organisms, such as fish [1], rats [2], and human health [3]. A number of countries and regions, including China [4], San Francisco [5], Canada, and the European Union [6], have issued policies banning BPA in products in response to the adverse effects of BPA. However, in order to meet the market demand for products, various alternatives to BPA were widely used in industrial production. This includes bisphenol-S (4,4′-sulfonyldiphenol; BPS), which is widely used in the manufacture of epoxy resins [7], bisphenol-E (bis(4-hydroxyphenyl) ethane, BPE) for cyanate resin [8], bisphenol-F (bis(4-hydroxypheny)methane; BPF) for epoxy resin reinforced with nano polyanilines [9], and bisphenol-AF (4,4′-(hexafluoroisopropylidene) diphenol, BPAF) and bisphenol-Z (4,4′-(cyclohexane-1,1-diyl) diphenol, BPZ) for the manufacture of epoxy resins and polycarbonate plastics [10]. However, recent studies have shown that BPS, BPF, and other BPA analogues were widely detected in the water environment, and the pollution levels have an increasing trend [11,12]. In addition, current research studies have indicated that BPS, BPAF, and BPF can be adsorbed by aquatic organisms, and pose a serious threat to the whole ecosystem as BPA [13,14]. Research is still needed to better elucidate the environmental sources, distribution, and fate of bisphenol analogues (BPs).

Most of the existing research studies focus on the traditional solubility phase, which was subdivided from natural water bodies, according to the operationally defined limit (e.g., 0.7 or 1.0 µm). However, natural colloids (1.0 nm–1.0 µm) are ubiquitously present in the traditional solubility phase. Due to its small size and large specific surface areas, colloids may have significance in terms of the proportion of bound pollutants and their subsequent behavior and fate [15]. Previous studies reported that 76% of total organic carbon were contributed by colloidal organic carbon. These colloids are important sinks of BPs and pharmaceutical active compounds [11,16]. In addition, up to 50% of antibiotics and BPs were associated with natural colloids in surface water [11,17]. However, few studies on the distribution and toxicological characteristics of BPs in the soluble (<1 nm) and colloidal phases have been investigated. Further studies on the exact distribution of BPs between different particle size factions in the aquatic system are needed.

Qinhuai River is connected with Gucheng Lake, Shijiu Lake, and Yangtze River, which is the chief receiving water body of domestic wastewater. Due to the rapid economic growth and high population density, the water quality of the Qinhuai River was gradually deteriorating. To improve water environmental quality of Qinhuai River, a project of the Yangtze River Diversion was implemented. The route of water diversion is: the Yangtze River → the New Qinghuai River → the Qinhuai River → the Yangtze River. By the mean of the Yangtze River Diversion project, contaminants can be diverted and diluted, and the water flow can be promoted. However, it is noteworthy that the diversion is discharged into the Yangtze River, which may pose a threat to the ecological health of the river. In this study, 13 sampling sites were settled up along the route of water diversion and the Nanjing section of Yangtze River. The distribution of six BPs in multiple environmental media, including the soluble phase, the colloidal phase, and suspended particulate matter of surface water were investigated. Based on this knowledge, the environmental risk of BPs was evaluated to determine a potential implication to the ecosystem of the water diversion project.

## 2. Materials and Methods

### 2.1. Chemicals

Bisphenol contaminants including BPA, BPS, BPE, BPF, BPAF, and bisphenol Z (BPZ) were purchased from J&K Chemical Co., Ltd. (Shanghai, China). Methanol, formic acid, and ammonia were obtained from Merk Corporation (Darmstadt, Germany).

### 2.2. Sample Collection

**Figure 1:**
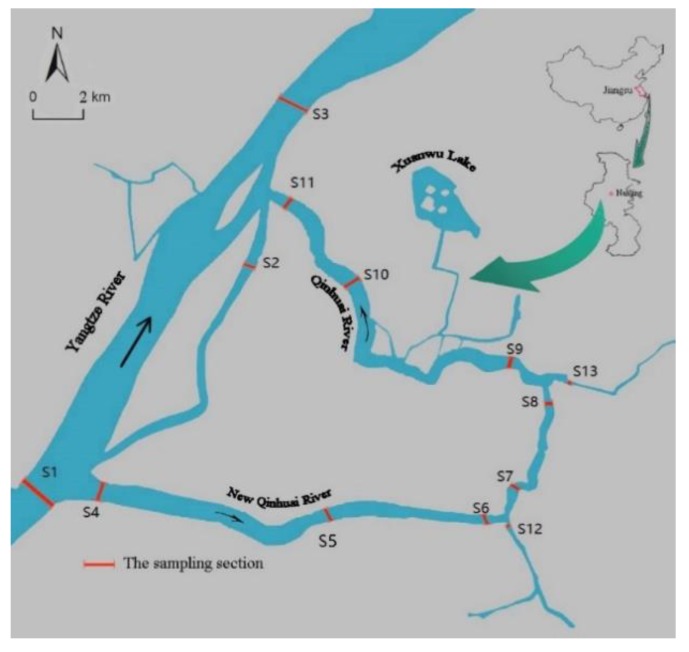
The locations of 13 sampling sites in the survey region from Nanjing, Jiangsu, China.

As shown in Figure 1, a total of 13 sampling sites were selected along the Yangtze River (S1–S3) and urban river (S4–S13). At each sampling site, 2 L of water sample was collected in triplicate on 15 July, 2018, according to the requirements for monitoring surface water [11]. The water samples collected were stored in containers containing dry ice, and quickly transported to the laboratory for further processing. According to the pretreatment method reported by a previous study [16], the water samples were divided into three parts (i.e., suspended particulate matter (SPM), colloidal, and soluble phase) by a vacuum filter unit and cross-flow cell, using 1 µm glass fiber filters and a 5 kDa polyether sulfone membrane, respectively.

### 2.3. Sample Extraction and Instrument Analysis

The methods for extraction and analysis of BPs in the soluble and colloidal phases were described in our previous study [11]. The solid-phase extraction (SPE) system was used to extract the exposure water samples through an Oasis hydrophilic lipophilic balance^®^ (6 mL, 200 mg) cartridge (Waters, Massachusetts, USA), and 9 mL of methanol with 2% ammonia was used to elute. Based on the previous study [18], the extraction methods of BPs in SPM samples were developed. The SPM samples were extracted using 20 mL of mixture solvent of methanol/acetone (50:50 *v*/*v*) with sonication for 30 min, and then centrifuged at 4000 r/min for 10 min. The above steps were repeated two times. The supernatant was merged and concentrated to 2 mL. Then, the supernatant was added into 200 mL ultra-pure water, and further cleanup was done using the SPE method similar to the method of water samples.

Waters Acquity ultra-high performance liquid chromatograph coupled with a Waters Acquity Xevo TQ triple quadrupole mass spectrometer was used to identify and quantify the BPs with multiple response monitoring. The mobile phase consisted of 0.01% ammonium hydroxide and 100% acetonitrile. The column temperature, the injection volume, and the flow rate were 40 °C, 5 µL, and 0.3 mL/min, respectively. Other instrumental parameters of UPLC/MS/MS were shown in supplementary information (Tables S1 and S2).

### 2.4. Quality Assurance and Quality Control

In the pretreatment process of the field sample, solvent blank, standard, and procedure blank were run successively to check the background of BPs. The limit of quantitative (LOQ) was used to evaluate sensitivity of the method. The LOQs of BPs in water samples and SPM samples are 0.53–11.1 ng/L and 0.25–1.5 ng/g, respectively, and the linear range is 1–200 ng/L (*R*^2^ > 0.991). The recovery rates of BPs in water samples and SPM samples are 70.3~108% and 73.5~110% (Table S3), respectively. The relative standard deviation is less than 20%. Detailed information was presented in supplementary information.

### 2.5. Parameter Measurement and Statistical Analysis

Using the risk quotient (RQ) of algae, daphnia spp. and fish conduct an ecological risk assessment of BPs detected in the traditional solubility phase [19]. According to the estrogen equivalent factor (EEF), 17 β-oestradiol equivalency quantity (EEQ) was calculated based on the measured concentrations of BPs [16]. The results were described using mean, median, and concentration ranges. The detailed information was shown in supplementary information.

## 3. Results and Discussion

### 3.1. Soluble Phase of Surface Water

**Figure 2:**
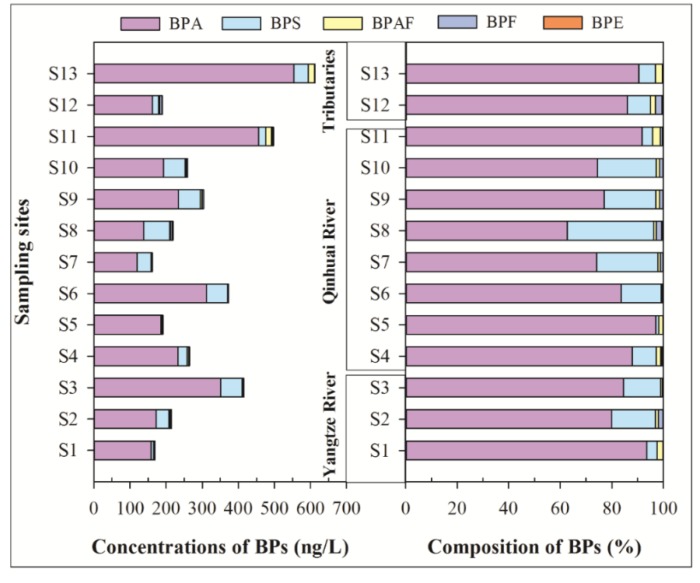
Concentrations and composition of bisphenol analogues in the soluble phase of surface water.

Table 1 shows the concentrations of BPs in surface water, including min-max, mean, and median values. In the six target BPs, only five were detected in the soluble phase, with a detection frequencies of 53.9–100%, while no BPZ was detected. BPA was the predominant compound (mean concentration: 253 ng/L), which is followed by BPS (39.2 ng/L) > BPAF (5.10 ng/L) > BPF (2.20 ng/L) > BPE (0.83 ng/L). As shown in Figure 2, the total concentrations of detected BPs (ΣBPs) are from 161 to 613 ng/L (mean concentration: 300 ng/L). Spatially, the tributary Yunliang River (S13) had the highest mean concentrations of ΣBPs, which is followed by the Qinhuai River outlet (S11), downstream of the Yangtze River (S3), and Qinhuai River. The Yunliang River as an urban receiving river, receives effluent of a sewage treatment plant (STP, Sewage treatment capacity: 200,000 m^3^/d) [16,20], overflow water of rainwater, and some untreated domestic sewage, which may be the main reason for high ΣBPs in S13. The lowest ΣBPs were found upstream of the Yangtze River, which ensure the availability of water quality of the Yangtze River Diversion. Compared to pollution levels upstream of the Yangtze River, the concentrations of ΣBPs downstream of the Yangtze River (S3) and the Qinhuai River outlet (S11) are 2.45 and 2.94 times higher, respectively. These results suggest that the water diversion project from the Yangtze River to the urban river reduced the concentrations of bisphenols in the aquatic environment of the urban river. However, it could also increase the ecological risk of BPs downstream of the Yangtze River.

In terms of monomer composition, the compositions of BPs in the soluble phase at each sampling site are generally similar. The dominant BP was BPA with a mean composition of 83.4%, followed by BPS (13.5%), BPAF (1.8%), and BPF (1.0%) (Figure 2). In a river-lake system of the Taihu Lake basin [11], the mean contribution rates of BPA, BPS, and BPF were 81.8%, 12.5%, and 4.7%, respectively, which were similar to our results. The pollution levels of BPA in sampling sites S3, S6, S11, and S13 were above 300 ng/L, while BPS in S3, S6, S8, S9, S10, and S13 were higher than 40 ng/L, which were similar with the Taihu Lake basin [11]. Among the alternatives to BPA, BPS was the most abundant alternative to BPA in the Nanjing section of the Yangtze River and Taihu Lake basin [21], which were clearly different from the Pearl River Delta, where BPF has 78.8% of a contribution rate as the main BP. These results suggest that BPS is being widely used in the Yangtze River basin.

### 3.2. Colloidal Phase of Surface Water

**Figure 3:**
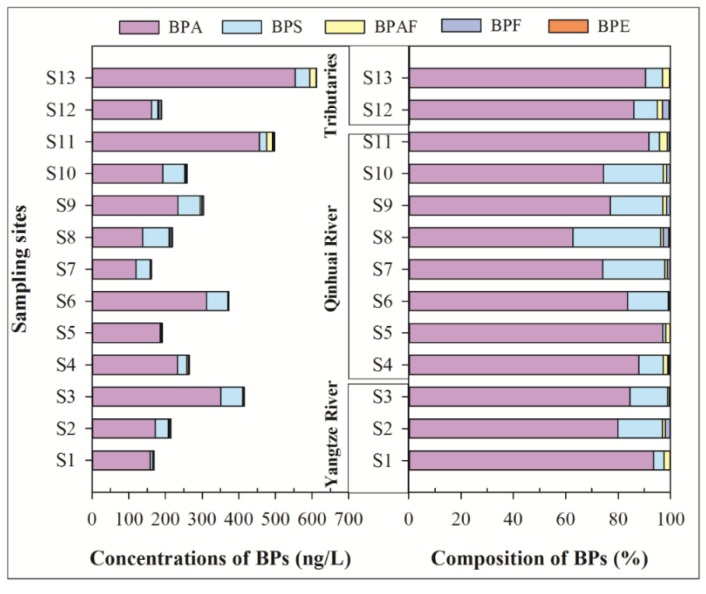
Concentrations and composition of BPs in the colloidal phase of surface water.

Colloids, as an important sink of emerging contaminants, is widely existing in an aquatic environment [22], which can cause several negative influences on the growth of an aquatic organism [23,24]. As shown in Figure 3, five of six BPs in the colloidal phase were widely detected with detection rates of 53.8–100%. Among them, the detection rates of BPA, BPAF, and BPS were 100%. The mean concentrations of ΣBPs were from 5.19 ng/L (site S1) to 77.2 ng/L (site S4), which were significantly lower than the soluble phase. This indicates that the biological availability of BPs was high. Similar to the monomer distribution of BPs in the soluble phase, BPA remains the primary BP, which is followed by BPS, BPAF, BPF, BPE, with mean concentrations of 27.4, 4.21, 1.12, 0.34, and 0.25 ng/L. These results were consistent with the Taihu Lake basin, where BPA and BPS were the dominant BPs [11]. In terms of monomer composition, the compositions of BPs in the colloidal phase were generally similar to that in the soluble phase. The mean contribution rate of BPA is 80.6%, which is followed by BPS (13.6%), BPAF (4.0%), BPF (1.2%), and BPE (0.6%). This was also similar to that of monomer distribution of BPs in the Taihu Lake basin [11].

### 3.3. Suspended Particulate Matter of Surface Water

**Figure 4:**
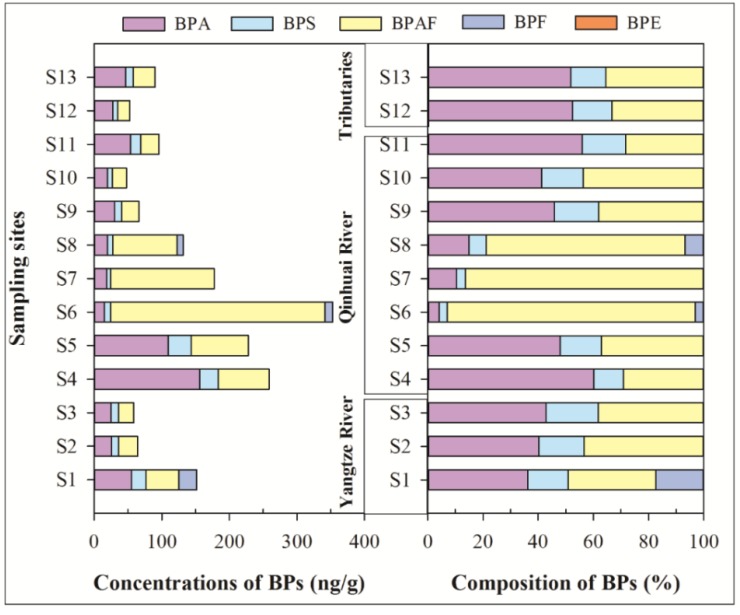
Concentrations and composition of BPs in suspended particulate matter of surface water.

Figure 4 presents the concentrations’ composition of BPs in the SPM samples. In the SPM, four of the six BPs were detected, and detection frequencies were 23.1–100%. Neither BPZ nor BPE was detected in all of the sampling sites. The mean concentrations of ΣBPs were from 47.5 ng/g to 353 ng/g. Among them, BPAF was the predominant BP, with a mean concentration of 46.7 ng/g, which is followed by BPA (38.8 ng/g), BPS (12.4 ng/g), and BPF (2.10 ng/g). In SPM samples of the Taihu Lake basin, the concentrations of BPA were from no detection (ND) to 877 ng/g dw (mean: 76.8 ng/g dw) [25], which were comparable to that of our study and the Yangtze River (Nanjing section). It ranged from ND to 364 µg/g (mean concentration 51.8 µg/g) [18]. However, only a few research studies were the focus on the other BPs’ residues in SPM.

The compositions of BPs in the SPM phase have been given in Figure 4. In contrast to the soluble and colloidal phases, BPAF exhibited relatively higher concentrations, where the mean contribution of BPAF to the ΣBPs (46.7%) was much higher than that in the soluble phase (1.8%) and colloidal phase (4.0%). The mean contribution of BPA to the ΣBPs (38.8%) in the SPM phase was lower than that in the soluble phase (83.4%) and colloidal phase (80.6%), which may be due to its powerful binding SPM capability because of the relatively high *K*_ow_ of BPAF. The mean contributions of BPAF in S6, S7, and S8 reached more than 72%. The three sites were located downstream of Jiangning STP, which may be related to the contribution variation of BPAF.

### 3.4. Partitioning Among Suspended Particulate Matter, Colloidal, and Soluble Phases of Surface Water

**Figure 5:**
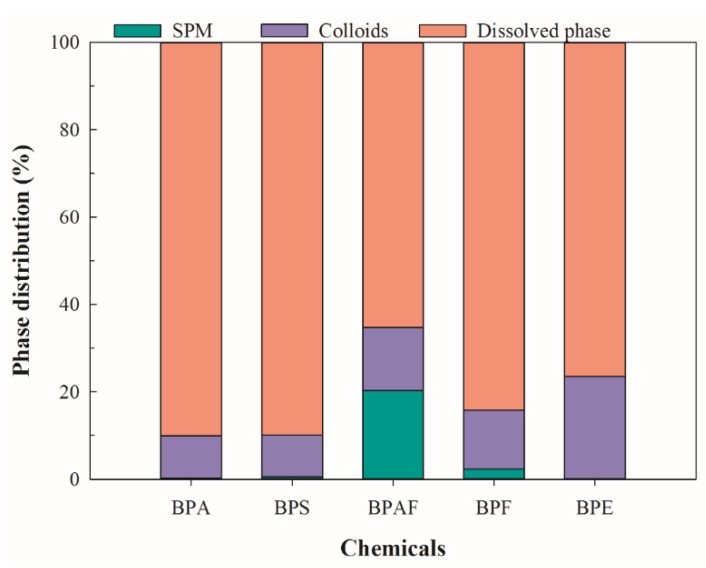
The BPs’ distribution in the SPM, soluble, and colloidal phases of surface water.

In order to assess the potential importance of particulate matter to environmental behavior of BPs in the aquatic environment, Figure 5 calculated the proportions of adsorption contribution of SPM and colloids to BPs. On average, 90.0% of BPA, 89.9% of BPS, 65.3% of BPAF, 84.2% of BPF, and 76.5% of BPE were found in the soluble phase. These findings were similar to those reported in the Pearl River [26], where between 24.4% and 94.1% of BPA was detected in the soluble phase. These results sugget that the majority of BPs exist in a dissolved state in surface water. In the colloidal phase, 9.7% of BPA, 9.6% of BPS, 14.4% of BPAF, 13.5% of BPF, and 23.5% of BPE were associated with colloids, which were covered in the results reported by Gong et al. [26], where colloid-bound BPs ranged from 3.6% to 52.4% for BPA, and 16.7% to 63.1% for 4-nonylphenol (log*K*_ow_ = 4.48). Si et al. found that 9.5% of BPS, 10.9% of BPA, 25.2% of BPF, and 50.4% of BPAF were bound with colloids in surface water from the Taihu Lake basin (Wujin district) without taking SPM into account [11]. In the present study, 0.2% of BPA, 0.5% of BPS, 2.3% of BPF, and 20.3% of BPAF were bound with SPM, which were much lower than that of colloids with the exception of BPAF. The findings indicate that colloids were potential sinks for BPs, which might influence the environmental behaviors of BPs due to its ubiquity, abundance, and mobility in aquatic systems.

Information about the occurrence of BPs is mainly concentrated in the traditionally soluble phase, and the pollution levels of BPs in the soluble and colloidal phases are small. In the present study, the mean concentrations of BPs in the traditionally soluble phase were from 174 ng/L to 642 ng/L (mean concentration: 344 ng/L). The mean contribution rates of the traditionally soluble phase were 99.8% of BPA, 99.5% of BPS, 79.7% of BPAF, 97.7% of BPF, and 100% of BPE. The pollution levels in the traditionally soluble phase reported in surface water worldwide are shown in Table S4. Based on frequent detection of BPs (BPA, BPF, BPS, and BPAF), the total concentrations of BPs in our study area were similar to those from the Taihu Lake basin investigated in 2018 [11] and the Pearl River Delta investigated in 2015 [27], with the concentration ranges of 98.8–726 ng/L and 107–987 ng/L, respectively. However, the contrast between the composition of BPs by BPA and BPF between Taihu Lake basin/Yangtze River (Nanjing section) and Pearl River Basin (China)/Tamagawa River (Japan)/Han River (Korea) showed the clear difference in the use and discharge of BPs in these regions/countries [21,27]. BPA was still the predominant one in Taihu Lake basin and the Yangtze River (Nanjing section) with mean concentrations of 217 ng/L and 291 ng/L, respectively [11], while BPF was abundant in the Pearl River basin, the Han River, and several rivers and bays in Japan (mean concentrations > 630 ng/L) [27]. In addition, BPS was the main bisphenols (mean concentrations of 2174 ng/L) in some rivers and lakes in India [27]. Therefore, we can find that there are lots of differences in BPs’ concentrations and distributions in different regions.

### 3.5. BPs Flux and Environmental Implication

On the basis of flow rates and mean concentrations of BPs, the flux of BPs through the water diversion project into the Yangtze River were estimated. The total flow rate of the water diversion project was 1.5 × 10^8^ m^3^/month during July. Based on the mean concentrations of BPs measured in the surface water of sampling site S11, the flux of BPs was calculated as 75.6 kg/month for BPA, 3.05 kg/month for BPS, 2.75 kg/month for BPAF, 0.59 kg/month for BPF, and 0.36 kg/month for BPE through the water diversion project. The total flux of BPs was 82.4 kg/month through the water diversion project.

**Figure 6:**
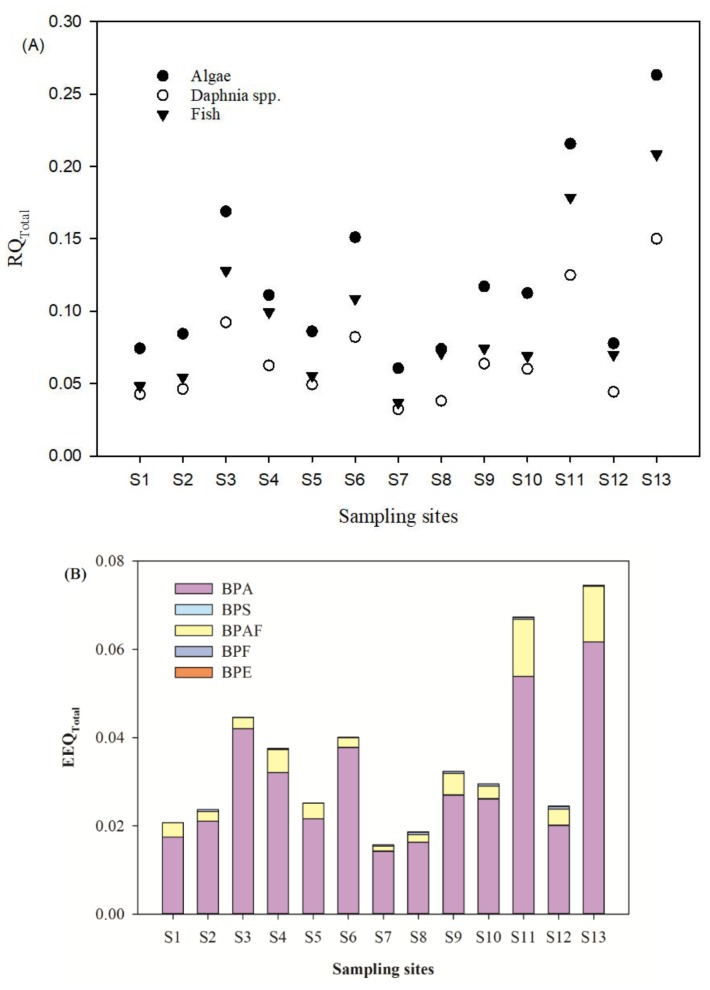
The RQ_Total_ (**A**) and EEQ_Total_ (**B**) of BPs for aquatic organisms in the surface water.

The increased emission and widespread presence of BPs in water bodies may have adverse effects on the ecosystem [28]. Screening level risk assessments of BPs were carried out based on the BPs’ concentrations in the traditionally soluble phase. The toxicity data of BPs were presented in Table S5 for the most sensitive aquatic organisms. Based on the evaluation criteria of risk levels (Low risk: 0.01 ≤ RQ < 0.1, medium risk: 0.1 ≤ RQ < 1, high risk: 1 ≤ RQ), the RQ values for detected BPs (except BPA) were mostly below 0.01, which suggests that low risk exists in the related aquatic organisms. The RQ values of BPA in most sampling sites exceeded 0.1 for algae, which indicates that medium risk for the growth status of *Selenastrum capricornutum* might exist [29]. Due to co-existence of BPs and their similar action patterns, the total risk quotient (RQ_Total_) of the detected BPs was evaluated to describe the worst-case scenario (Figure 6A). The RQ_Total_ of algae were from 0.067 to 0.274, daphnia spp. from 0.036 to 0.156, and fish from 0.041 to 0.236, respectively. In this study, fish was the most sensitive species, which was followed by daphnia spp. and algae. In general, the mixture risk contribution rate of each BP decreased in following order: BPA (87.4%) > BPE (5.9%) > BPAF (3.8%) > BPS (2.0%) > BPF (0.9%).

According to the EEF value in Table S5, 17 β-oestradiol equivalency quantity (EEQ) method was applied to calculate the estrogen activity of BPs. When the total EEQ (EEQ_Total_) > 1.0 ng E_2_/L, it was shown that the chemical could have a negative effect on the endocrine system of aquatic organisms. In our study, the results showed that EEQ_Total_ in the waters was 0.0157–0.0745 ng E_2_/L (Figure 6B). All the sampling points were lower than 1.0 ng E_2_/L. The contribution rate of BPA to estrogen activity was the highest, which was 86.2%. This is followed by BPAF (12.9%), BPF (0.8%), and BPS (0.1%). The higher environmental risk of BPs was found in sampling sites S11 and S13. These two sampling sites are located in the estuarine of the water diversion project into Yangtze River and the ravine stream into the water diversion project. These suggest that the flushing and dilution effects of the water diversion project exist for the mitigation of BPs contamination in the urban river. However, potential environmental risks transferring into the Yangtze River also need to be taken into consideration.

It must be noted that the toxicity estimate presented in this study does not reflect the overall ecotoxicological risk of the study area, which provides an assessment of the total ecotoxicological risk contribution of the detected BPs. In addition, with the prohibition of BPA, the concentration of BPs will continuously increase in the aquatic environment, and some BPs was detected in human urine, serum, and breast milk [30]. Long time exposure to BPs may have adverse effects on the development of the reproductive and nervous system for biota and humans [31,32,33]. Some scholars suggest that pregnant women and babies should avoid exposure to BPs by reducing the use of plastic products, canned food, personal care products, and thermal receipt papers [31].

## 4. Conclusions

The occurrence and distribution of six BPs in surface water were observed in the water diversion project. The results indicated that BPA, BPAF, BPS, and BPF were found to exist widely in SPM, colloidal and soluble phases, with the total concentration ranging from 47.5 to 353 ng/g, 5.19 to 77.1 ng/L, and 161 to 613 ng/L, respectively. In terms of medium partitioning, the majority of BPs exist in a dissolved state in surface water, which is followed by colloids and SPM adsorption. The mean distribution ratios of the three media were 0.7%, 9.9%, and 89.4% for the SPM, colloidal, and soluble phases, respectively. Among the detected BPs, BPA and BPS were the most abundant in the soluble and colloidal phases, while BPA and BPAF were the primary pollutants in the SPM. Particulate matter showed significant binding capacity for BPs, especially for colloids, which may further influence the environmental behaviors of BPs. The spatial distribution of BPs in surface water showed that BPs’ concentrations was high and environmental risks of BPs were found in the estuarine of the water diversion project into the Yangtze River, which may further affect the ecological security of the Yangtze River.

## Figures and Tables

**Table 1:** Concentrations of bisphenol analogues detected in the suspended particulate matter, colloidal phases of surface water.

Compounds	Soluble Phase (ng/L)				Colloidal Phase (ng/L)				SPM (ng/g)			
	Mean	Median	Range	DF ^a^ (%)	Mean	Median	Range	DF (%)	Mean	Median	Range	DF (%)
BPA	253	222	120–554	100	27.4	22.3	4.54–66.7	100	38.8	42.8	28.2–89.8	100
BPS	39.2	38.3	2.24–73.3	100	4.21	4.20	0.14–10.2	100	12.6	14.5	2.78–19.0	100
BPAF	5.10	3.47	1.50–16.2	100	1.12	0.97	0.12–2.47	100	46.7	38.1	28.2–89.8	100
BPF	2.20	1.90	0.00–4.76	61.5	0.35	0.33	0.00–0.82	69.2	2.10	0.00	0.00–17.3	23.1
BPE	0.83	0.94	0.00–2.12	53.8	0.25	0.23	0.00–1.11	53.8	0.00	0.00	0.00–0.00	0.00

^a^ DF: detection frequency.

## Supplementary Materials

Supplementary information of this article can be found online at https://www.mdpi.com/1660-4601/16/18/3296/s1. Table S1: The mobile phase compositions of the separation methods. Table S2: The six BPs of the optimized MS/MS parameters. Table S3: Recovery values obtained for the three independent concentrations of spiked quality control samples. Table S4: Concentrations of BPs reported as mean (median) and minimum-maximum observed globally. Table S5: The BPs’ aquatic toxicity data of the most sensitive aquatic species.

## References

[1] Suzuki N., Kambegawa A., Hattori A. (2003). Bisphenol A influences the plasma calcium level and inhibits calcitonin secretion in goldfish. Zool. Sci..

[2] Liu H., Huang Q., Sun H., Li J., Lin Q., Wu H., Liu C. (2019). Effects of separate or combined exposure of nonylphenol and octylphenol on central 5-HT system and related learning and memory in the rats. Ecotoxicol. Environ. Saf..

[3] Xu X., Qin J., Wei Y., Ye S., Shen J., Yao Y., Ding B., Shu Y., He G., Chen H. (2019). Heterogeneous activation of persulfate by NiFe_2−x_Co_x_O_4_-RGO for oxidative degradation of bisphenol A in water. Chem. Eng. J..

[4] Jin H., Zhu L. (2016). Occurrence and partitioning of bisphenol analogues in water and sediment from Liaohe River basin and Taihu Lake, China. Water Res..

[5] Chen D., Kannan K., Tan H., Zheng Z., Feng Y., Wu Y., Widelka M. (2016). Bisphenol Analogues Other Than BPA: Environmental Occurrence, Human Exposure, and Toxicity—A Review. Environ. Sci. Technol..

[6] Negev M., Berman T., Reicher S., Balan S., Soehl A., Goulden S., Diamond M.L. (2018). Regulation of chemicals in children’s products: How US and EU regulation impacts small markets. Sci. Total Environ..

[7] Liang W., Zhao B., Zhao P., Zhang C., Liu Y. (2017). Bisphenol-S bridged penta (anilino) cyclotriphosphazene and its application in epoxy resins: Synthesis, thermal degradation, and flame retardancy. Polym. Degrad. Stabil..

[8] Thunga M., Lio W., Kessler M. (2011). Adhesive repair of bismaleimide/carbon fiber composites with bisphenol E cyanate ester. Compos. Sci. Technol..

[9] Zhang X., He Q., Gu H., Colorado H., Wei S., Guo Z. (2013). Flame-Retardant Electrical Conductive Nanopolymers Based on Bisphenol F Epoxy Resin Reinforced with Nano Polyanilines. ACS Appl. Mater. Inter..

[10] Song S., Duan Y., Zhang T., Zhang B., Zhao Z., Bai X., Sun H. (2019). Serum concentrations of bisphenol A and its alternatives in elderly population living around e-waste recycling facilities in China: Associations with fasting blood glucose. Ecotoxicol. Environ. Saf..

[11] Si W., Cai Y., Liu J., Shen J., Chen Q., Chen C., Ning L. (2019). Investigating the role of colloids on the distribution of bisphenol analogues in surface water from an ecological demonstration area, China. Sci. Total Environ..

[12] Luo J., Zhang Q., Cao M., Wu L., Cao J., Fang F., Li C., Xue Z., Feng Q. (2018). Ecotoxicity and environmental fates of newly recognized contaminants-artificial sweeteners: A review. Sci. Total Environ..

[13] Ding Z.M., Jiao X.F., Wu D., Zhang J.Y., Chen F., Wang Y.S., Huang C.J., Zhang S.X., Li X., Huo L.J. (2017). Bisphenol AF negatively affects oocyte maturation of mouse in vitro through increasing oxidative stress and DNA damage. Chem.-Biol. Interact..

[14] Yan Z., Liu Y., Yan K., Wu S., Han Z., Guo R., Chen M., Yang Q., Zhang S., Chen J. (2017). Bisphenol analogues in surface water and sediment from the shallow Chinese freshwater lakes: Occurrence, distribution, source apportionment, and ecological and human health risk. Chemosphere.

[15] Persson Y., Shchukarev A., Oberg L., Tysklind M. (2008). Dioxins, chlorophenols and other chlorinated organic pollutants in colloidal and water fractions of groundwater from a contaminated sawmill site. Environ. Sci. Pollut. R..

[16] Liu J., Lu G., Xie Z., Zhang Z., Li S., Yan Z. (2015). Occurrence, bioaccumulation and risk assessment of lipophilic pharmaceutically active compounds in the downstream rivers of sewage treatment plants. Sci. Total Environ..

[17] Cheng D., Liu X., Zhao S., Cui B., Bai J., Li Z. (2017). Influence of the natural colloids on the multi-phase distributions of antibiotics in the surface water from the largest lake in North China. Sci. Total Environ..

[18] Liu Y.H., Zhang S.H., Ji G.X., Wu S.M., Guo R.X., Cheng J., Yan Z.Y., Chen J.Q. (2017). Occurrence, distribution and risk assessment of suspected endocrine-disrupting chemicals in surface water and suspended particulate matter of Yangtze River (Nanjing section). Ecotoxicol. Environ. Saf..

[19] Backhaus T., Karlsson M. (2014). Screening level mixture risk assessment of pharmaceuticals in STP effluents. Water Res..

[20] Liu J., Dan X., Lu G., Shen J., Wu D., Yan Z. (2018). Investigation of pharmaceutically active compounds in an urban receiving water: Occurrence, fate and environmental risk assessment. Ecotoxicol. Environ. Saf..

[21] Liu Y., Zhang S., Song N., Guo R., Chen M., Mai D., Yan Z., Han Z., Chen J. (2017). Occurrence, distribution and sources of bisphenol analogues in a shallow Chinese freshwater lake (Taihu Lake): Implications for ecological and human health risk. Sci. Total Environ..

[22] Yan C.X., Nie M.H., Yang Y., Zhou J.L., Liu M., Baalousha M., Lead J.R. (2015). Effect of colloids on the occurrence, distribution and photolysis of emerging organic contaminants in wastewaters. J. Hazard. Mater..

[23] Bai Y.Y., Zheng M.F., Zheng A.R., Chen D., Liu C.L. (2007). Effects of Nature Colloids on the Growth of Nature Bacteria Community. J. Xiamen Univ. (Nat. Sci.).

[24] Wang F., Zhu G.W., Xu H., Qin B.Q. (2009). The bioeffect of natural colloids on the growth of *Microcystis aeruginosa* in Lake Taihu, China. China Environ. Sci..

[25] Liu D., Liu J.N., Guo M., Xu H.Z., Zhang S.H., Shi L.L., Yao C. (2016). Occurrence, distribution, and risk assessment of alkylphenols, bisphenol A, and tetrabromobisphenol A in surface water, suspended particulate matter, and sediment in Taihu Lake and its tributaries. Mar. Pollut. Bull..

[26] Gong J., Huang Y.D., Huang W., Ran Y., Chen D.Y. (2016). Multiphase partitioning and risk assessment of endocrine disrupting chemicals in the Pearl River, China. Environ. Toxicol. Chem..

[27] Yamazaki E., Yamashita N., Taniyasu S., Lam J., Lam P.K.S., Moon H.B., Jeong Y., Kannan P., Achyuthan H., Munuswamy N. (2015). Bisphenol A and other bisphenol analogues including BPS and BPF in surface water samples from Japan, China, Korea and India. Ecotoxicol. Environ. Saf..

[28] Yan Z., Yang H., Dong H., Ma B., Sun H., Pan T., Jiang R., Zhou R., Shen J., Liu J. (2018). Occurrence and ecological risk assessment of organic micropollutants in the lower reaches of the Yangtze River, China: A case study of water diversion. Environ. Pollut..

[29] Debenest T., Gagné F., Petit A.N., André C., Kohli M., Blaise C. (2010). Ecotoxicity of a brominated flame retardant (tetrabromobisphenol A) and its derivatives to aquatic organisms. Comp. Biochem. Physiol. C Toxicol. Pharmacol..

[30] Song M.Y., Liang D., Liang Y., Chen M.J., Wang F.B., Wang H.L., Jiang G.B. (2014). Assessing developmental toxicity and estrogenic activity of halogenated bisphenol A on zebrafish (Danio rerio). Chemosphere.

[31] Wu L.H., Zhang X.M., Wang F., Gao C.J., Chen D., Palumbo J., Guo Y., Zeng E. (2018). Occurrence of bisphenol S in the environment and implications for human exposure: A short review. Sci. Total Environ..

[32] Cao L.Y., Ren X.M., Li C.H., Zhang J., Qin W.P., Yang Y., Wan B. (2017). Bisphenol AF and Bisphenol B exert higher estrogenic effects than Bisphenol A via G protein-coupled estrogen receptor pathway. Environ. Sci. Technol..

[33] Moreman J., Lee O., Trznadel M., David A., Kudoh T., Tyler C. (2017). Acute Toxicity, Teratogenic, and Estrogenic Effects of Bisphenol A and Its Alternative Replacements Bisphenol S, Bisphenol F, and Bisphenol AF in Zebrafish Embryo-Larvae. Environ. Sci. Technol..

