# Supplementary material for: Occurrence, Distribution and Ecological Risk of Bisphenol Analogues in the Surface Water from a Water Diversion Project in Nanjing, China
Source: Int J Environ Res Public Health. 2019 Sep 7;16(18):3296. doi: 10.3390/ijerph16183296 (PMC6765785; doi:10.3390/ijerph16183296)
Supplement: Supplementary file 1 [file ijerph-16-03296-s001.pdf]

Contents

2.3 Sample extraction and instrument analysis

2.4 Quality assurance and quality control

2.5 Parameter measurement and statistical analysis

Table S1. The mobile phase compositions of the separation methods.

Table S2. The six BPs of the optimized MS/MS parameters.

Table S3. Recovery values obtained for the three independent concentrations of spiked quality control samples.

Table S4. Concentrations of BPs reported as mean (median) and minimum-maximum observed globally.

Table S5. The BPs' aquatic toxicity data of the most sensitive aquatic species.

2.3 Sample extraction and instrument analysis

Other instrumental parameters of UPLC/MS/MS were showed in Table S1 and Table S2.

Table S1

**Time (min)**	**Composition of the mobile phase (%)**	
Negative mode	Eluent A2 (0.01%Ammonium hydroxide)	Eluent B1 (Acetonitrile)
0	90	10
0.25	90	10
3.00	10	90
4.00	10	90
4.01	90	10
5.00	90	10

Table S2

**Compound**	**Retention time/min**	**Parent ion (m/z)**	**Daughter ions(m/z)**	**Dewll time(s)**	**Conevol tage (v)**	**Collision energy (v)**
Bisohenol F	2.74	199.10	93.10	0.042	30	20

Bisphenol E	2.89	213.10	105.10*<br>198.00*<br>/	0.042	40	20<br>22<br>/
Bisphenol A	3.03	227.10	133.00<br>212.00*	0.042	31	25<br>17
Bisphenol S	0.66	249.13	92.05<br>108.07*	0.161	42	30<br>24
Bisphenol Z	3.45	267.22	93.02<br>173.17*	0.042	56	32<br>30
Bisphenol AF	3.25	335.23	197.10<br>265.16*<br>419.92*	0.042	32	25<br>22<br>40

* represents quantification ion.

2.4 Quality assurance and quality control

The strict QA/QC protocol was used to detect the extraction efficiency of BPs in the water sample. The performance of the analytical method was evaluated in terms of linearity, limit of detection (LOD) and limit of quantitative (LOQ) and recovery rate. When analyzing each group of samples, running solvent, standar, and process blanks in turn to check background BPs, peak identification, and quantification. Respectively, LOD and LOQ were determined to be the minimum detectable amounts of the analyte with signal-to-noise (S/N) of 3 and 10. Through injecting different concentrations of the standard solutions (seven-point calibration curve) into the 1.0-200 ng/mL range (R^2> 0.998), the linearity of the target compounds was studied. In order to evaluate the recovery, the spiked samples were prepared using ordinary surface water samples. Prior to use, surface water samples used for recovery test were analyzed to detect the presence of BPs. The recovery data for BPs had been corrected to take into account the fact that the average blank peak area of BPs was subtracted from the average peak area of the other recovery points. Six separate chromatographic runs were performed on each of the two concentration levels. The analysis of the reagent blanks (n=3) showed that the analysis system and glassware did not contain BPs.

Table S3

Compound	Recovery [%] (n = 6)				Quantitation limits (n = 3)	
	Water sample		SPM samples		Water	SPM
	10 ng/L	100 ng/L	10 ng/g	100 ng/g	(ng/L)	(ng/g)
BPF	83.0±12.4	97.8±10.2	82.4±13.1	90.2±10.0	11.10	1.50
BPE	88.4±7.2	92.3±10.7	75.7±6.3	82.0±10.4	1.83	0.50
BPA	91.0±8.1	108±9.9	102±10.8	110±11.5	2.60	0.25
BPS	80.3±4.0	91.4±16.3	79.3±9.3	84.7±6.5	0.53	0.30
BPZ	70.3±5.6	85.6±10.0	73.5±7.4	80.2±7.1	7.07	1.00
BPAF	93.1±16.3	96.2±8.0	82.2±11.4	92.3±10.1	1.70	0.30

2.5 Parameter measurement and statistical analysis

Using the risk quotient (RQ) to evaluate the risk assessment of the target compounds in the urban water. The RQ_s was calculated as Eq (1).

$$RQ = \frac{MEC}{PNEC} = \frac{MEC}{EC50 \text{ or } LC50/f} \quad (1)$$

MEC and PNEC are measured environmental concentrations and predicted no-effect concentrations. According to the REACH guidance document, in order to estimate PNEC based on toxicity data when only short-term/acute toxicity data EC_{50} or LC_{50} is available, PNEC is calculated by the EC50 or LC50 that divides the safety factor (*f*) 1000. Once a long-term/chronic NOEC value of one, two or three nutritional levels is available, using the *f* of the 100, 50, or 10 (ECHA, 2008). PNEC is derived from chronic and acute toxicity data in the literature and is 100 or 1000 *f* in our study. Table 3 provides PNEC calculations for algae, daphnia and fish.

Calculation of the oestrogen equivalent concentration (EEQ) of a chemically determined mixture is based on all measured xenoestrogens with a known oestrogen equivalency factor (EEF; Table 3), as shown in the following equation (Eq. (2)). When EEQ_{Total} >1.0 ng E_2/L, the contaminants are thought to affect the endocrine systems of organisms in the water bodies. So EEQ is also used to assess the risk of BPs to human health.

$$EEQ_{Total} = \Sigma EEQ_i = \Sigma(C_i \times EEF_i) \quad (2)$$

The C_i refers to the compound *i* with a concentration of C in the traditional dissolved phase. EEQ_{Total} is the total estradiol equivalent and EEF_i is the estradiol equivalent.

Table S4

Sampling locations	Time	Concentrations (ng/L) in traditionally soluble phase				Reference
		BPA	BPS	BPF	BPAF	
Study area	2018 [a] (07)	290 (244) 133-576	43.6(42.1) 5.87-83.5	2.53 (2.23) ND-5.44	6.68 (4.51) 1.62-17.8	This study
Wujin district	2018 (08)	217 (157) 73.5-678	60.5(32.1) 7.80-319	7.13 (4.61) 1.14-40.1	2.70 (2.05) 0.30-17.7	[1]
Taihu Lake	2013 (09)	8.5 (7.9) 4.2-14	6.0 (2.0) 0.28-67	0.83 (0.5) ND-5.6	0.28 (0.2) 0.13-1.1	[2]
Taihu Lake	2015 (05)	9.7 (7.3) 3.9-33.2	2.6 (0.94) 0.32-27.3	1.24 (1.1) 0.5-3.28	0.27 (0.1) 0.06-2	[3]
Taihu Lake	2015 (11)	92.6 (53.2) 28-565				[4]
Taihu Lake	2016 (04)	97 28-560	120 4.5-1600	140 ND-1600	8.2 0.7-23	[5]
Taihu Lake	2016 (11)	25.7 (23.8) 19.4-68.5	15.9 (6.6) 41.-157	78 (30) 25.6-723	114 (111) 110-140	[6]
Luoma Lake	2016 (04)	86 49-110	21 ND-94	6.8 3.5-14	17 12-84	[5]
Liaohe River	2013 (09)	47 (29) 5.9-141	14 (8.9) 0.22-52	ND[b]	1.9 (1.0) 0.5-9.6	[2]
Hunhe River	2013 (09)	40 (42) 4.4-107	11 (8.4) 0.61-46	ND	2.4 (0.94) 0.61-11	[2]
Pearl River	2013 (07)	73 (73) ND-98	135 (135) ND-135	773 (757) 448-1110	ND	[7]
West River	-2014 (03)	43 (43) ND-43	ND	64 (64) ND-105	ND	
20 source water China	2017 (11)	12.8 (10.5) ND-34.9	1.1 (0.4) ND-5.2	2.2 (ND) ND-12.6	3.0 (0.1) ND-10.8	[8]
Several Rivers, Bay (Japan)	2013 (07) -2014 (03)	104 ND-431	5.3 ND-15	638 ND-2850	ND	[7]
Several Rivers (Korea)		105.7 1.0-272	41 ND-42	633 ND-1300	ND	
Several Rivers, Lake (India)		551 ND-1950	2174 ND-7200	91.5 ND-289	ND	

[a] Year (Month).

[b] ND: not detected.

Table S5

Compound	Non-target organism	Test Endpoint	Toxicity data (mg/L)	PNEC (ng/L)	Reference	EEF Ref. [17]
BPA	Algae	72h-EC50	2.2 (Growth)	2200	[9]	1.07×10^{-4}
	Daphnia spp.	48h-EC50	3.9 (Immobility)	3900	[10]	
	Fish	48h-EC50	3.6 (Pigmentation)	3600	[11]	
BPS	Algae	96h-EC50	6.9	6900	[13] [a]	1.06×10^{-6}
	Daphnia spp.	48h-EC50	55 (Immobility)	55000	[14]	
	Fish	72 hpf-EC50	155 (Mortality)	155000	[15]	
BPF	Algae	72h-IC50	22.1 (Growth)	22100	[11]	1.08×10^{-4}
	Daphnia spp.	21d-NOEC	0.84 (Reproduction)	8400	[11]	
	Fish	48h-EC50	1.1 (Pigmentation)	1100	[11]	
BPAF	Algae	72h-IC50	3.0 (Growth)	3000	[11]	7.23×10^{-4}
	Daphnia spp.	21d-NOEC	0.23 (Reproduction)	2300	[11]	
	Fish	72hpf-EC50	0.92 (Mortality)	920	[15]	
BPE	Daphnia spp.	48h-EC50	18	18000	[14]	5.92×10^{-5}
	Fish	EC50	0.0579	57.9	[16]	

[a] The toxicity data was calculated from the ecological structure activity relationships (ECOSAR) model.

Reference

1. Si, W., Cai, Y., Liu, J., Shen, J., Chen, Q., Chen, C., Ning, L., 2019. Investigating the role of colloids on the distribution of bisphenol analogues in surface water from an ecological demonstration area, China. Science of the Total Environment. 673, 699-707.

2. Jin, H., Zhu, L., 2016. Occurrence and partitioning of bisphenol analogues in water and sediment from Liaohe River basin and Taihu Lake, China. Water Research. 103, 343-351.

3. Wang, Q., Chen, M., Shan, G., Chen, P., Cui, S., Yi, S., Zhu, L., 2017. Bioaccumulation and biomagnification of emerging bisphenol analogues in aquatic organisms from Taihu Lake, China. Science of the Total Environment 598, 814-820.

4. Liu, D., Liu, J.N., Guo, M., Xu, H.Z., Zhang, S.H., Shi, L.L., Yao, C., 2016. Occurrence, distribution, and risk assessment of alkylphenols, bisphenol A, and tetrabromobisphenol A in surface water, suspended particulate matter, and sediment in Taihu Lake and its tributaries. Marine Pollution Bulletin. 112, 142-150.

5. Yan, Z., Liu, Y., Yan, K., Wu, S., Han, Z., Guo, R., Chen, M., Yang, Q., Zhang, S., Chen, J., 2017. Bisphenol analogues in surface water and sediment from the shallow Chinese freshwater lakes:

Occurrence, distribution, source apportionment, and ecological and human health risk. Chemosphere. 184, 318-328.

6. Liu, Y., Zhang, S., Song, N., Guo, R., Chen, M., Mai, D., Yan, Z., Han, Z., Chen, J., 2017. Occurrence, distribution and sources of bisphenol analogues in a shallow Chinese freshwater lake (Taihu Lake): Implications for ecological and human health risk. Science of the Total Environment. 599, 1090-1098.

7. Yamazaki, E., Yamashita, N., Taniyasu, S., Lam, J., Lam, P. K.S., Moon, H.B., Jeong, Y., Kannan, P., Achyuthan, H., Munuswamy, N., Kannan, K., 2015. Bisphenol A and other bisphenol analogues including BPS and BPF in surface water samples from Japan, China, Korea and India. Ecotoxicology and Environmental Safety. 122, 565-572.

8. Zhang, H., Zhang, Y., Li, J., Yang, M., 2019. Occurrence and exposure assessment of bisphenol analogues in source water and drinking water in China. Science of the total Environment. 655, 607-613.

9. Debenest, T., Gagné, F., Petit, A. N., André, C., Kohli, M., Blaise, C., 2010. Ecotoxicity of a brominated flame retardant (tetrabromobisphenol A) and its derivatives to aquatic organisms Comp. Comparative Biochemistry and Physiology Part C: Toxicology & Pharmacology. 152. 407-412.

10. Staples, C.A., Dome, P.B., Klecka, G.M., Oblock, S.T., Harris, L.R., 1998. A review of the environmental fate, effects, and exposures of bisphenol A. Chemosphere. 36, 2149-2173.

11. Tišler, T., Krel, A., Gerželj, U., Erjavec, B., Dolenc, M. S., Pintar, A., 2016. Hazard identification and risk characterization of bisphenols A, F and AF to aquatic organisms. Environmental Pollution. 212, 472-479.

13. US Environmental Protection Agency. 2011. Estimation Program Interface EPI, Suite, Version Environmental Protection Agency, Office of Pollution Prevention and Toxic's, Washington, DC, USA.

14. Chen, M.Y., Ike, M., Fujita, M., 2002. Acute toxicity, mutagenicity, and estrogenicity of bisphenol-A and other bisphenols. Environmental Toxicology. 17, 80-86.

15. Moreman, J., Lee, O., Trznadel, M., David, A., Kudoh, T., Tyler, C.R., 2017. Acute toxicity, teratogenic, and estrogenic effects of bisphenol A and its alternative replacements bisphenol S, bisphenol F, and bisphenol AF in zebrafish embryo-larvae. Environmental Science & Technology. 51, 12796-12805.

16. Owczarek, K., Kudlak, B., Simeonov, V., Mazerska, Z., Namiesnik, J., 2018. Binary Mixtures of Selected Bisphenols in the Environment: Their Toxicity in Relationship to Individual Constituents. Molecules. 23, 3226.

17. Ruan, T., Liang, D., Song, S., Song, M., Wang, H., Jiang, G., 2015. Evaluation of the in vitro estrogenicity of emerging bisphenol analogs and their respective estrogenic contributions in municipal sewage sludge in China. Chemosphere. 124, 150-155.
